# Supplementary material for: Seasonal mixed layer depth shapes phytoplankton physiology, viral production, and accumulation in the North Atlantic
Source: Nat Commun. 2021 Nov 17;12:6634. doi: 10.1038/s41467-021-26836-1 (PMC8599477; doi:10.1038/s41467-021-26836-1)
Supplement: Supplementary file 1 — Supplementary Info (PDF version) [file 41467_2021_26836_MOESM1_ESM.pdf]

SUPPLEMENTARY INFORMATION

Seasonal mixed layer depth shapes phytoplankton physiology, viral production, and accumulation in the North Atlantic

Ben Diaz[1], Ben Knowles[1†], Christopher T. Johns[1], Christien P. Laber[1‡], Karen Grace V. Bondoc[1], Liti Haramaty[1], Frank Natale[1], Elizabeth L. Harvey[2], Sasha J. Kramer[3,4], Luis Bolanos[5x], Daniel P. Lowenstein[6], Helen Fredricks[6], Jason Graff[7], Toby Westberry[7], Kristina D. A. Mojica[7#], Nils Haëntjens[8], Nicholas Baetge[9], Peter Gaube[10], Emmanuel Boss[8], Craig A. Carlson[9], Michael J. Behrenfeld[7], Benjamin A.S. Van Mooy[6], and Kay D. Bidle[1*]

Affiliations

[1] Department of Marine and Coastal Science, Rutgers University, New Brunswick, NJ 08901, USA

[2] Department of Biological Sciences, University of New Hampshire, Durham, NH 03824, USA

[3] Interdepartmental Graduate Program in Marine Science, University of California, Santa Barbara, Santa Barbara, CA, United States

[4] Earth Research Institute, University of California, Santa Barbara, Santa Barbara, CA, United States

[5] Department of Microbiology, Oregon State University, Corvallis, OR, United States

[6] Department of Marine Chemistry and Geochemistry, Woods Hole Oceanographic Institution, Woods Hole, MA 02543, USA

[7] Department of Botany and Plant Pathology, Oregon State University, Corvallis, OR 97331, USA

[8] School of Marine Sciences, University of Maine, Orono, ME 04469, USA

[9] Department of Ecology, Evolution, and Marine Biology, Marine Science Institute, University of California Santa Barbara, Santa Barbara, CA 93106, USA

[10] Applied Physics Laboratory, University of Washington, Air-Sea Interaction and Remote Sensing Department, Seattle, WA, USA

[†] Current address: Department of Ecology and Evolutionary Biology, University of California, Los Angeles, Los Angeles, CA, United States

[‡] Current address: Centre for Ecology and Evolution in Microbial Model Systems, Linnaeus University, SE-39182 Kalmar, Sweden

[#] Current address: Division of Marine Science, School of Ocean Science and Engineering, The University of Southern Mississippi, Stennis Space Center, MS, United States

[x] Current Address: School of Biosciences, University of Exeter, Exeter, UK

*Corresponding author
bidle@marine.rutgers.edu

Supplementary Table 1-2
Supplementary Figures 1-14
Supplementary References

Supplementary Table 1. Summary of biomarkers used in this study and their associated organismal sources. "X" and blue filling denotes organisms that were included in our analysis methods; "-" denotes organisms that were likely excluded due to their size range (Caspase, metacaspase, SYBR Gold) or lack of chlorophyll autofluorescence (ROS, Comp. membranes); Reference numbers provided used a similar or the exact same method as our study in each of the four types of organisms presented. "NA" denotes that no reference was found with that biomarker and organism group. *- inner diameter of tubing used to remove water from Niskin bottles

Reagent	Abbreviation	Biomarker	Size range sampled	Eukaryotic phytoplankton	Prokaryotic phytoplankton	Eukaryotic heterotrophs	Prokaryotic heterotrophs
CM-H_2DCFDA	ROS	Intracellular reactive oxygen species	1-20 µm	X[1–3]	-[4]	-[5]	-[6]
SYTOX Green	Comp. Membrane	Compro-mised cells membranes/ dead cells	1-20 µm	X[1,2,7,8]	-[9]	-[10]	-[11]
	TAG	Tri-acylglycerol, Storage lipid	0.2 µm < 9.5 mm*	X[12]	X[NA*]	X[13]	X
	Ox PC/ PC	Oxidized membrane lipids	0.2 µm < 9.5 mm*	X[14]	X[NA]	X[15]	X[NA]
IETD-AFC	Caspase activity	Cysteine-dependent, aspartate-specific protease activity	1.2 µm < 9.5 mm*	X[16]	-[16]	X[NA]	-[17]
VRPR-AMC	Metacaspase activity	Cysteine-dependent protease activity	1.2 µm < 9.5 mm*	X[16]	-[16]	X[NA]	-[18]
SYBR Gold	Virus L^{-1}	dsDNA, dsRNA viruses	0.05-0.2 µm	-[19]	-[19]	-[20]	-[19]
SYBR Gold	Bacteria L^{-1}	Bacteria	0.2-0.5 µm	-[19]	X[21]	-[20]	X[21]
Alcian Blue	TEP L^{-1}	Transparent exopolymer particles	>0.45 µm	X[22–26]	X[26]	X[NA]	X[27]
	DOC	Dissolved Organic Carbon	>0.7 µm		-		-

Supplementary Table 2. Spline types used to construct General Additive Modeling of Stratification. Spline types were optimized via AIC criterion (see Methods). TP = thin plate spline; CS = cubic spline; CC= cyclic cubic spline.

	Generalized Additive Modeling, spline type			
	W.Tran.	Acc	Clim	Dec
Phytoplankton cells L^{-1}	TP	TP	TP	CS
Bacteria L^{-1}	CC	CS	TP	TP
Virus L^{-1}	TP	CC	TP	TP
DOC (µM)	TP	TP	CS	CS

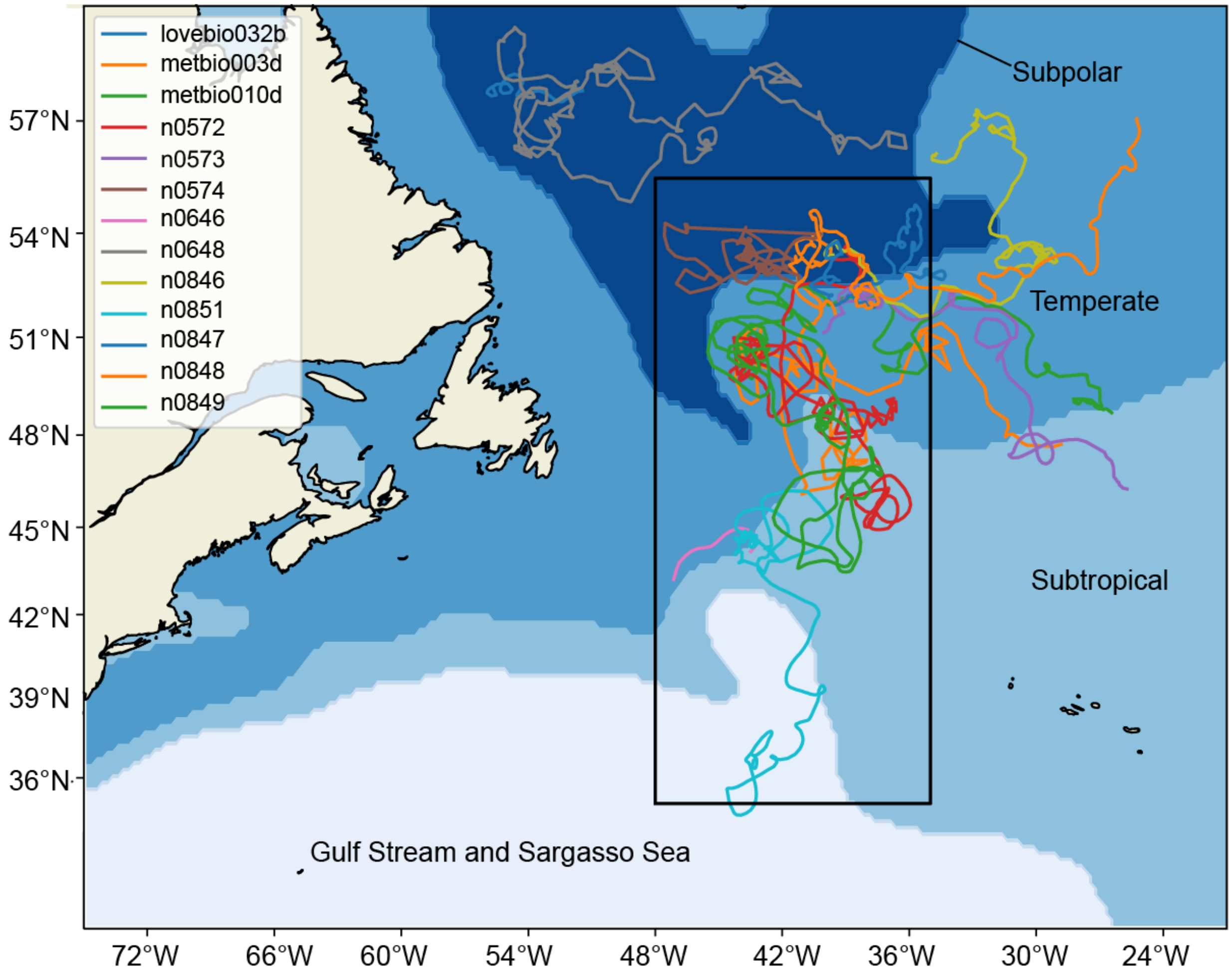

Supplementary Figure 1. Tracks of profiling floats used in the study. Different profiling floats deployed prior to and during NAAMES expeditions are represented by different colors and are overlaid onto subregions (Gulf Stream and Sargasso Sea, Subtropical, Temperate and Subpolar; as defined by Della Penna and Gaube[28]). The black rectangle denotes the study area used for binning mixed layer depths reported in Figure 1b. Profiling floats with an "n" prefix (ex. "n0847) were deployed during one of the four NAAMES expeditions; other profiling floats (ex. "lovebio003d") were deployed as part of other projects and provided additional contextual data for subregions within the NAAMES study area[29]. Water is colored by subregions and profiling float tracks are colored by float ID's.

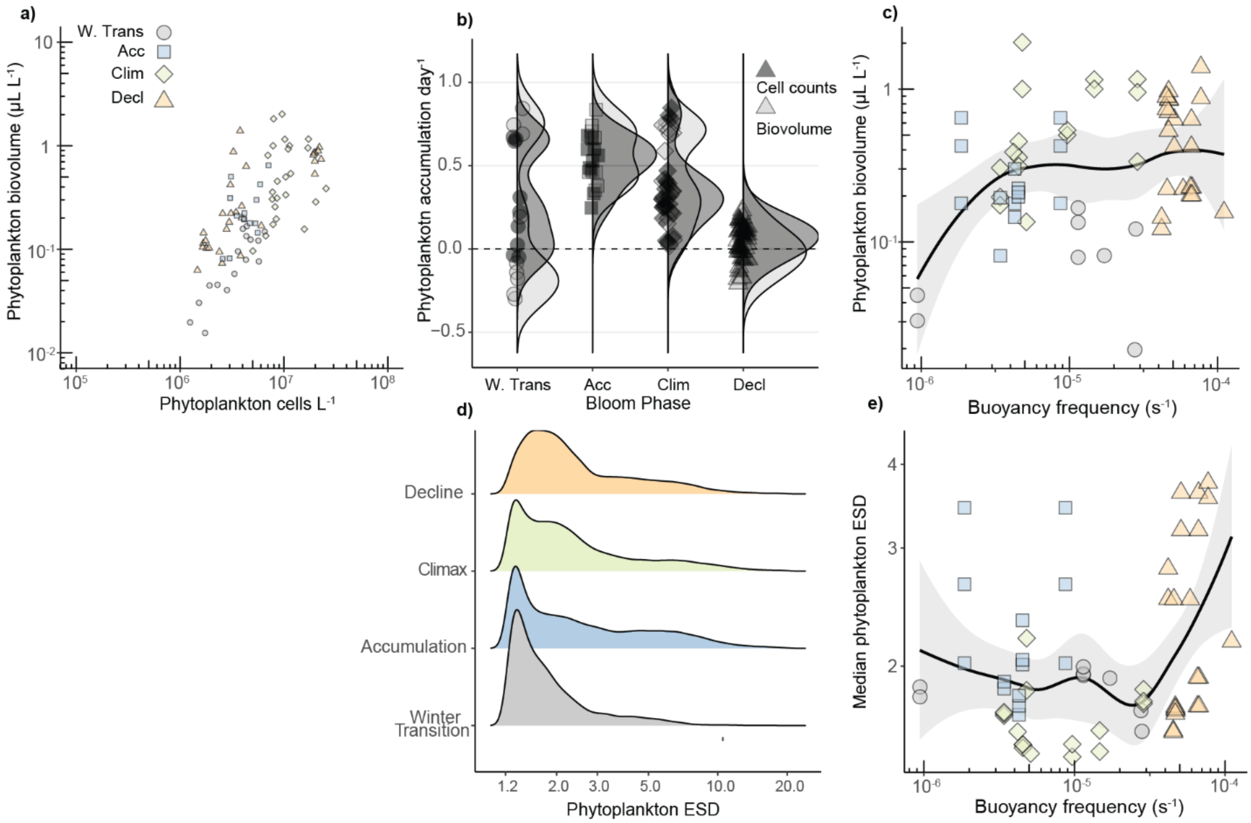

Supplementary Figure 2. Biovolume-based phytoplankton accumulation dynamics and relationship to water column stratification. a) Relationship between *in situ* phytoplankton concentration and corresponding biovolume across phases during NAAMES (indicated by color coded symbols). b) Comparison of biovolume- and cell concentration-based accumulation rates from on-deck incubations (dark grey data correspond to that presented in Fig. 1d). c) Phytoplankton biovolume as a function of water column stratification (expressed as buoyancy frequency; s^{-1}). Higher buoyancy frequencies to the right of the plot represent more stratification. d) Phytoplankton equivalent spherical diameter (ESD, μm) at depths corresponding to 40% surface irradiance. Note the shift to increased cell sizes during decline phase. (e) Median phytoplankton ESD (μm) as a function of water column stratification (expressed as buoyancy frequency; s^{-1}). A LOESS line of best fit with shaded 95% confidence interval is shown for data across all phases in c) and e). Significant differences within each cruise were not detected between different methods for calculating accumulation rates in b), as assessed using Kruskal Wallis rank-sum test with Bonferroni corrections for multiple comparisons, p<.05. Data in b) and d) are contoured with ridgeline smoothing to represent the distribution of biovolume accumulation rates biovolume and ESD, respectively, within each seasonal phase. Points or frequencies in a), c), d), e) are colored by bloom phase. Points in panels a), c), and e) are shaped by bloom phase. Exact p values from b) can be found in Source Data file.

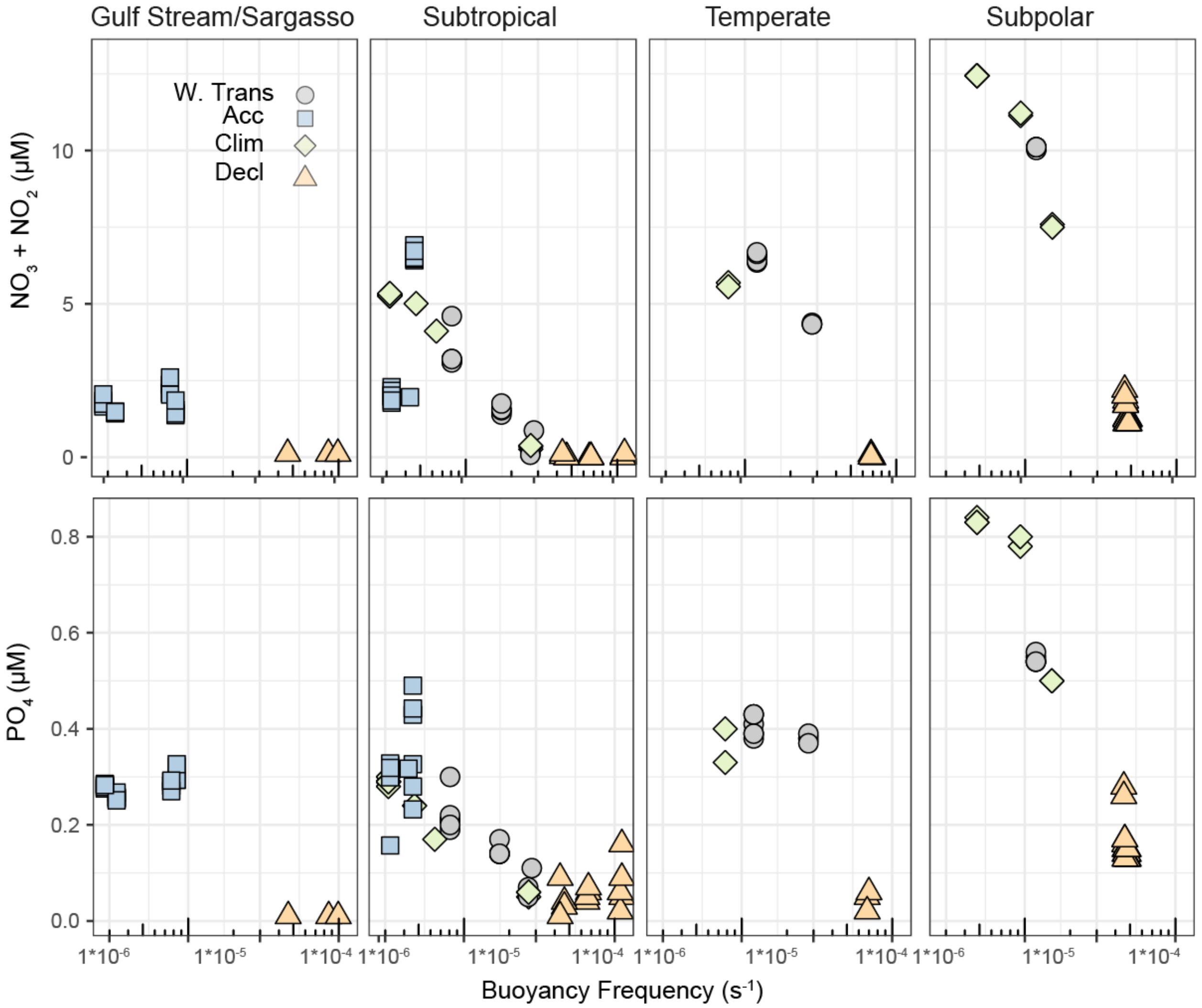

Supplementary Figure 3: Nutrient concentrations within the mixed layer as a function of stratification. In situ, mixed layer concentrations of nitrate +nitrite (NO_3 + NO_2) and phosphate (PO_4) at NAAMES stations during bloom phases. Stations are broken out by water type designations of each panel column (Gulf Stream and Sargasso Sea, Subtropical, Temperate, and Subpolar, as defined by Della Penna and Gaube 2019[28]and mapped in Fig. 1a and Supplementary Fig. 1). Point shape and color correspond to bloom phase (W. Tran = Winter Transition; Acc = Accumulation; Clim = Climax; Decl = Decline).

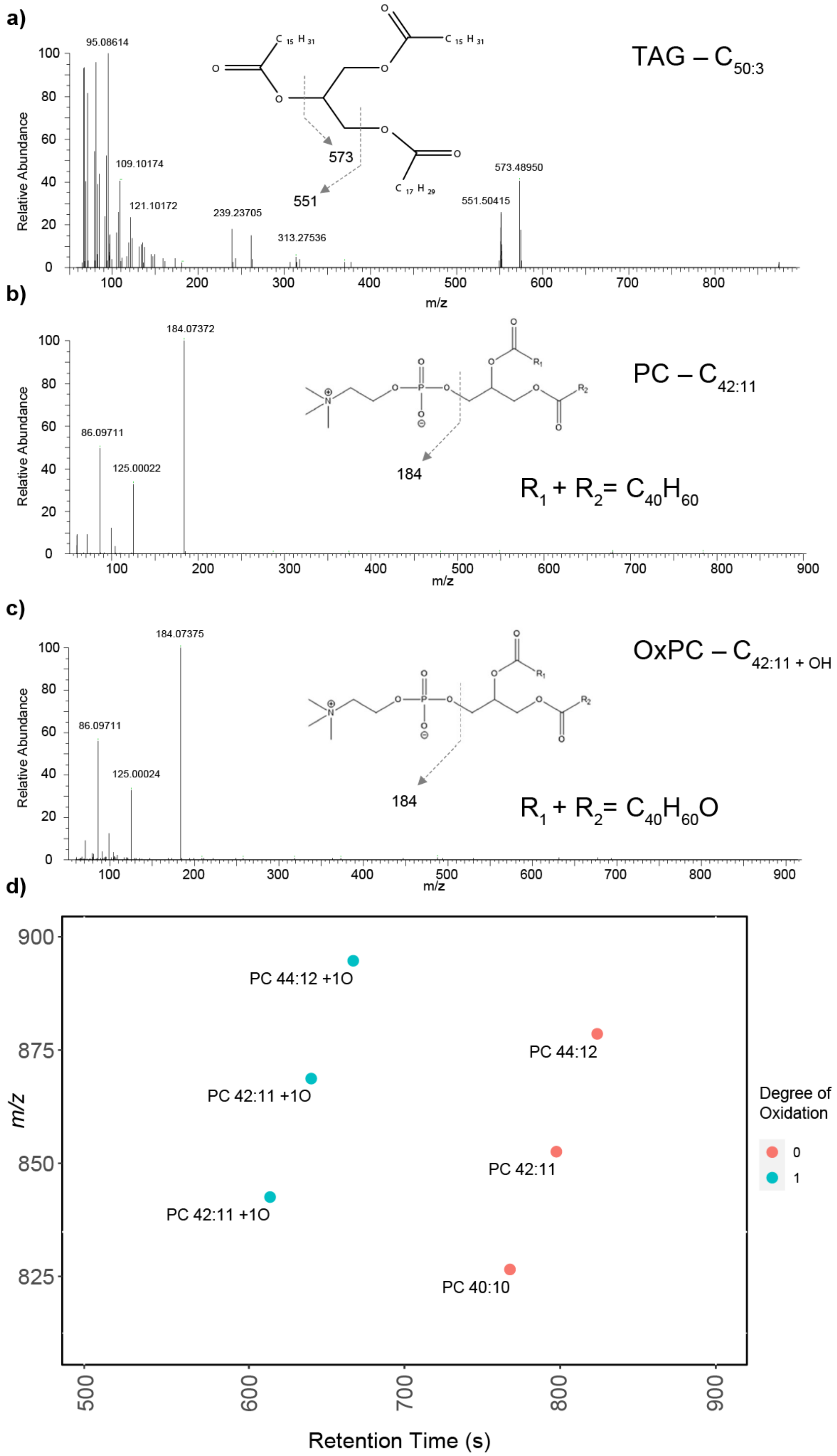

Supplementary Figure 4. Representative mass spectra, mass:charge ratios, and retention times

used to confirm lipid assignment by LOBSTAHS software. MS^2 spectra of a) $[M+NH_4]^+$ at *m/z* 846.75452 of TAG – $C_{50:3}$ ($C_{53}H_{96}O_6$); b) $[M+H]^+$ at *m/z* 852.55378 of PC – $C_{42:11}$ ($C_{50}H_{78}NO_8P$); c) $[M+H]^+$ at *m/z* 868.54870 of OxPC – $C_{42:11\ +\ OH}$ ($C_{50}H_{78}NO_9P$); identified in seawater samples collected during NAAMES cruises, and peak groups aligned using xcms software. PC = phosphatidylcholine; OxPC = oxidized phosphatidylcholine; TAG = triacylglycerol. d) HPLC retention time vs. *m/z* plot of phosphatidylcholine peak group centroids showing diagnostic shifts in both *m/z* and retention times due to oxidation. These were used to discern the relative amounts of OxPC, colored by 0 and 1 degrees of oxidation.

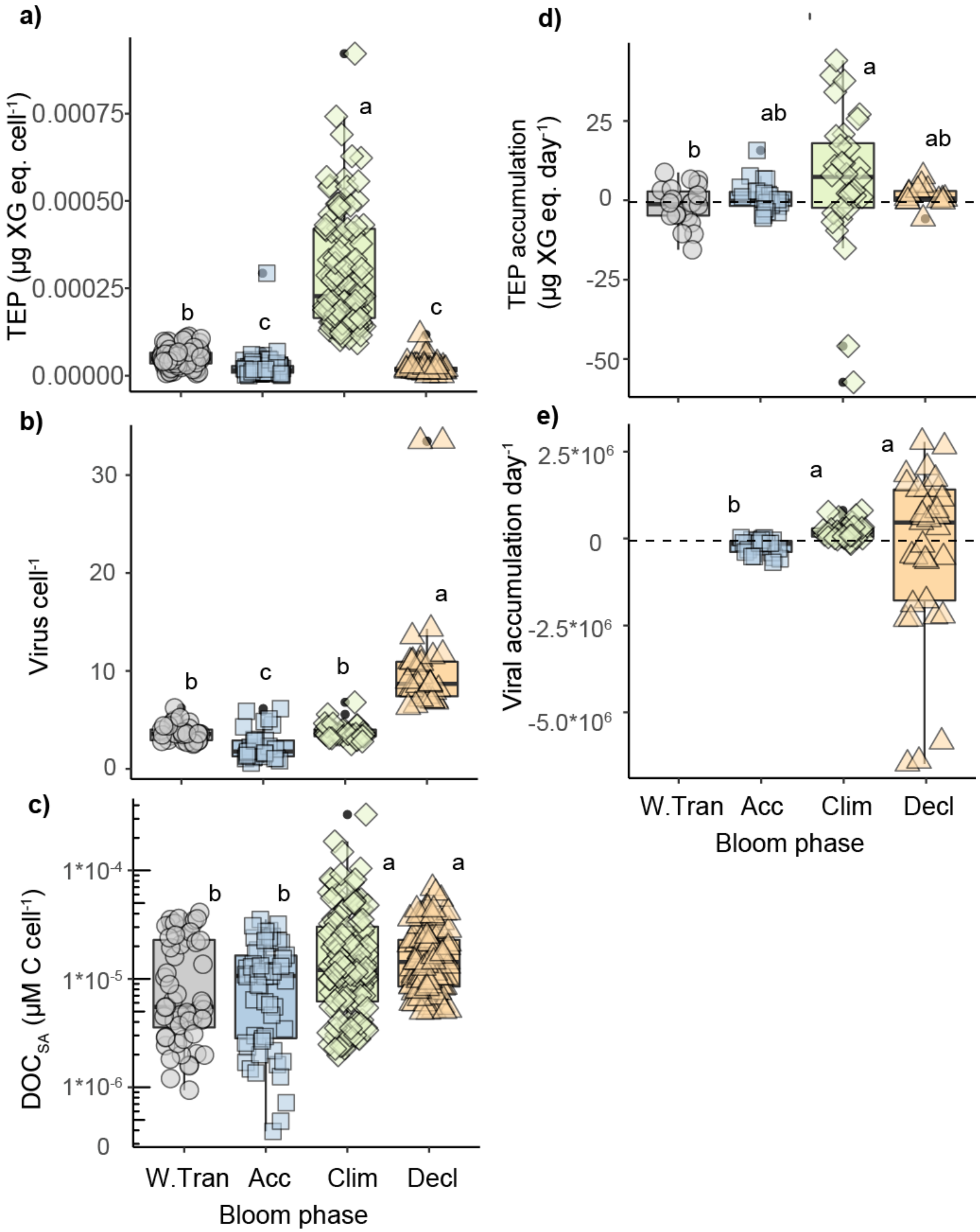

Supplementary Figure 5. Cell-normalized concentrations and accumulation of extracellular constituents. Cell (phytoplankton and bacteria)-normalized concentrations of a) transparent exopolymer particles (TEP, µg xanthan gum equivalent cell^{-1}), b) viruses cell^{-1}, and c) seasonally accumulated dissolved organic carbon (DOC_{SA}, µM carbon cell^{-1}) within the mixed

layer (40%, 20%, or 1% surface irradiance) for different bloom phases sampled during NAAMES (W.Tran = Winter Transition; Acc = Accumulation; Clim = Climax; Decl = Decline). Average accumulation (average change per day, see Methods) of d) Transparent exopolymers (μg xanthan gum equivalent L^{-1}) and e) viruses L^{-1} within the mixed layer for different phases sampled during NAAMES. Individual symbols are colored and shaped by bloom phase and represent biological replicates, taken within the mixed layer. Box plots represent the median value bounded by the upper and lower quartiles with whiskers representing the median + quartile*1.5. Different letters denote statistically significant groups ($p<0.05$, Kruskal-Wallis test with Dunn corrections for multiple comparisons). Intergroup comparisons with more than one letter denote no significant difference between the two groups. Number of biologically independent samples by bloom phase (from left to right) = 93, 76, 107, 55 (a), 32, 30, 36, 34 (b), 56, 58, 124, 117 (c), 22, 26, 32, 14 (d), 0, 24, 35, 31 (e). Exact p values can be found in Source Data file.

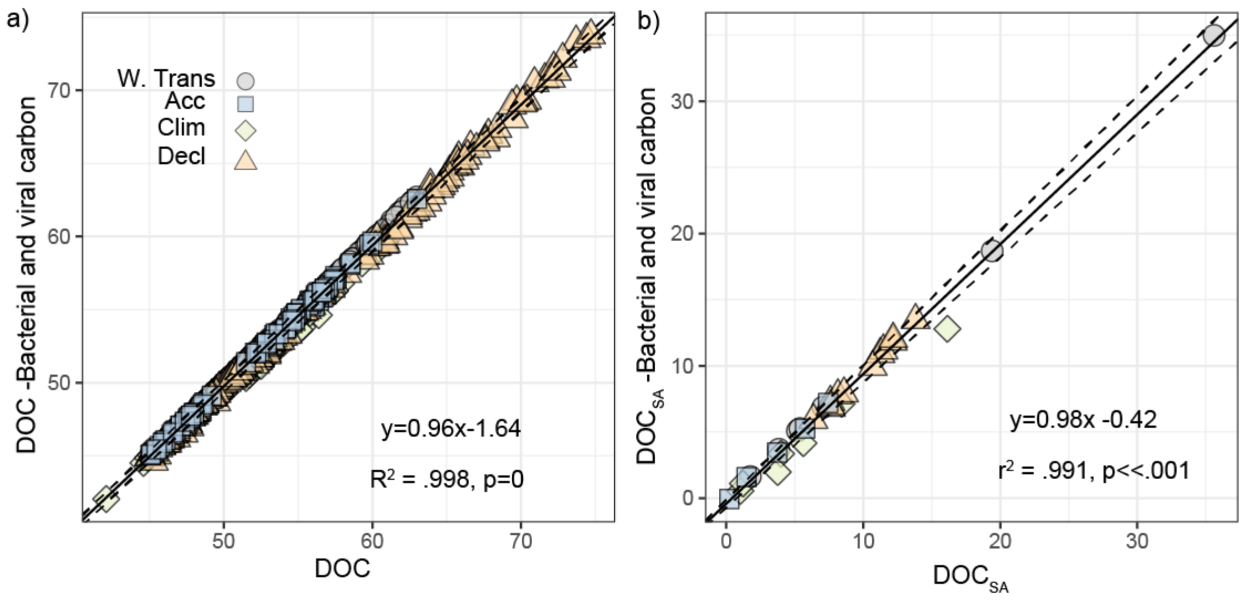

Supplementary Figure 6. Bacteria and virus pools make negligible contribution to total DOC and DOC_{SA} during the North Atlantic bloom. Relationships between a) dissolved organic carbon (DOC) and b) Seasonally accumulated (DOC_{SA})[30] with and without bacterial and viral contributions. A Model II linear regression (MA, two-tailed) is shown with the area between the dotted lines representing the 95%confidnce interval. Calculations of the contribution of bacterial and viral carbon to respective DOC pools used 60% of the total bacteria counted and 100% of the total viruses counted via flow cytometry (see Methods), at 12.430 and 0.231 fg per particle based on prior estimations[31,32], respectively. Note that the combined bacteria and virus pools have negligible contributions to DOC_{SA} as the microbe-corrected 95% confidence interval falls on the 1:1 line. Symbols are colored and shaped by bloom phase. Bloom phase abbreviations (bottom row): W. Tran = Winter transition, Acc = Accumulation, Clim = Climax, Decl = Decline.

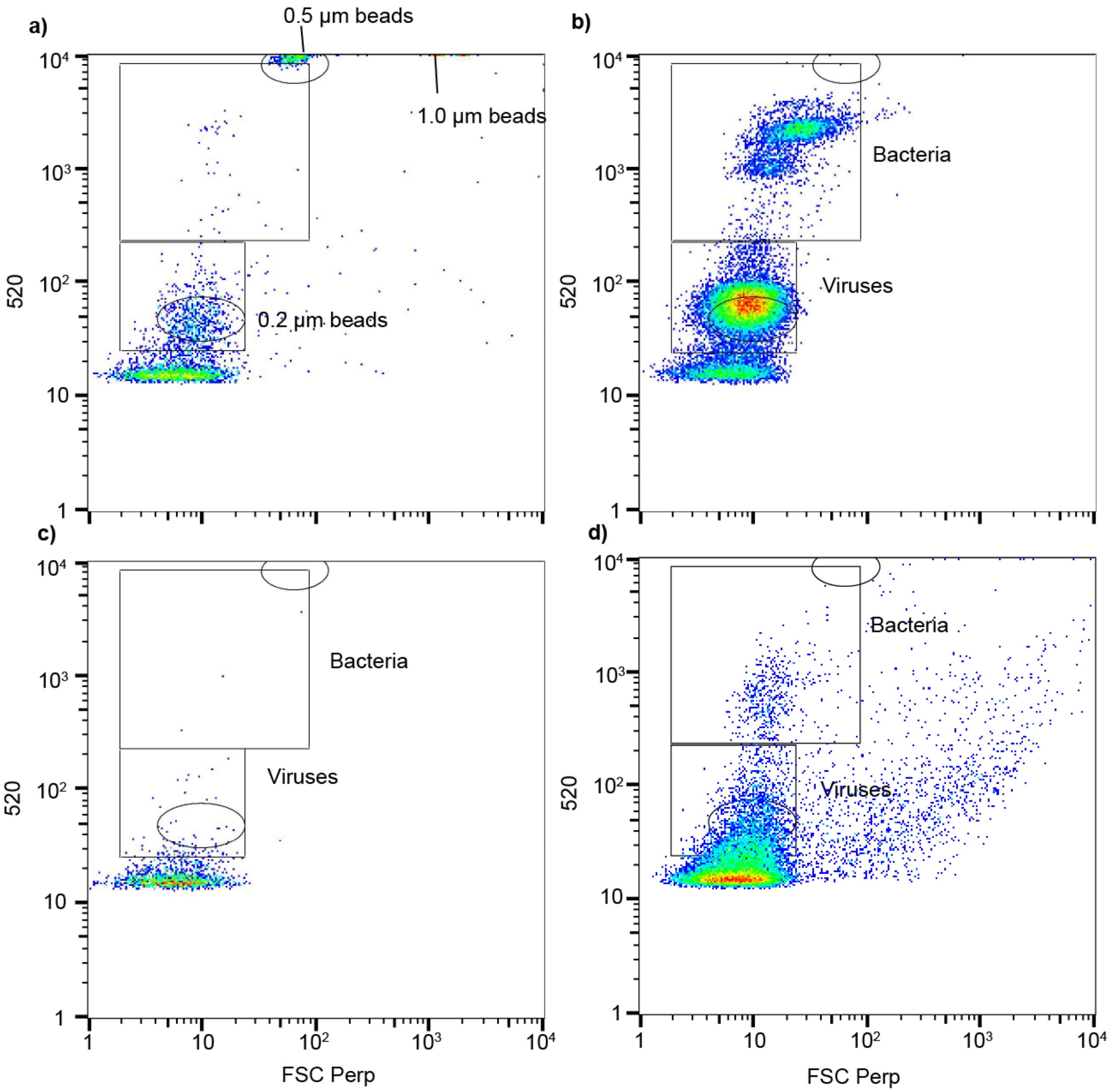

Supplementary Figure 7. Gating strategy used for determination of bacteria and virus-like particle/ virus concentrations via flow cytometry. False-color flow cytograms are shown for different sample types for forward scatter (FSC), a proxy for particle size, and green fluorescence (520 nm) via SYBR staining, which stains viral genomic DNA. a) Bead size standards (0.2, 0.5 and 1.0 µm) diluted in MQ water overlaid with gates used to enumerate bacterial and viruses. b) A 0.45 µm-filtered viral lysate of *Emiliania huxleyi CCMP*374 infected with *Emiliania huxleyi virus* 207 (EhV207) was used as a positive control to set bacteria and virus gates. Given EhV207 is ~180 nm in diameter, the virus gate was extended to include smaller viruses. c) Sample blank consisting of SYBR Gold and TE buffer (pH 8) heated to 80°C for 10 minutes. The median events mL^{-1} of five different blank samples was used as a representative blank. d) Example of a NAAMES field sample from the Decline phase. The particle counts falling within the “Viruses” and ”Bacteria” gates in blank sample c) were subtracted from all field samples to obtain the final concentrations. These virus-like particles are referred to as “viruses” in the rest of this text.

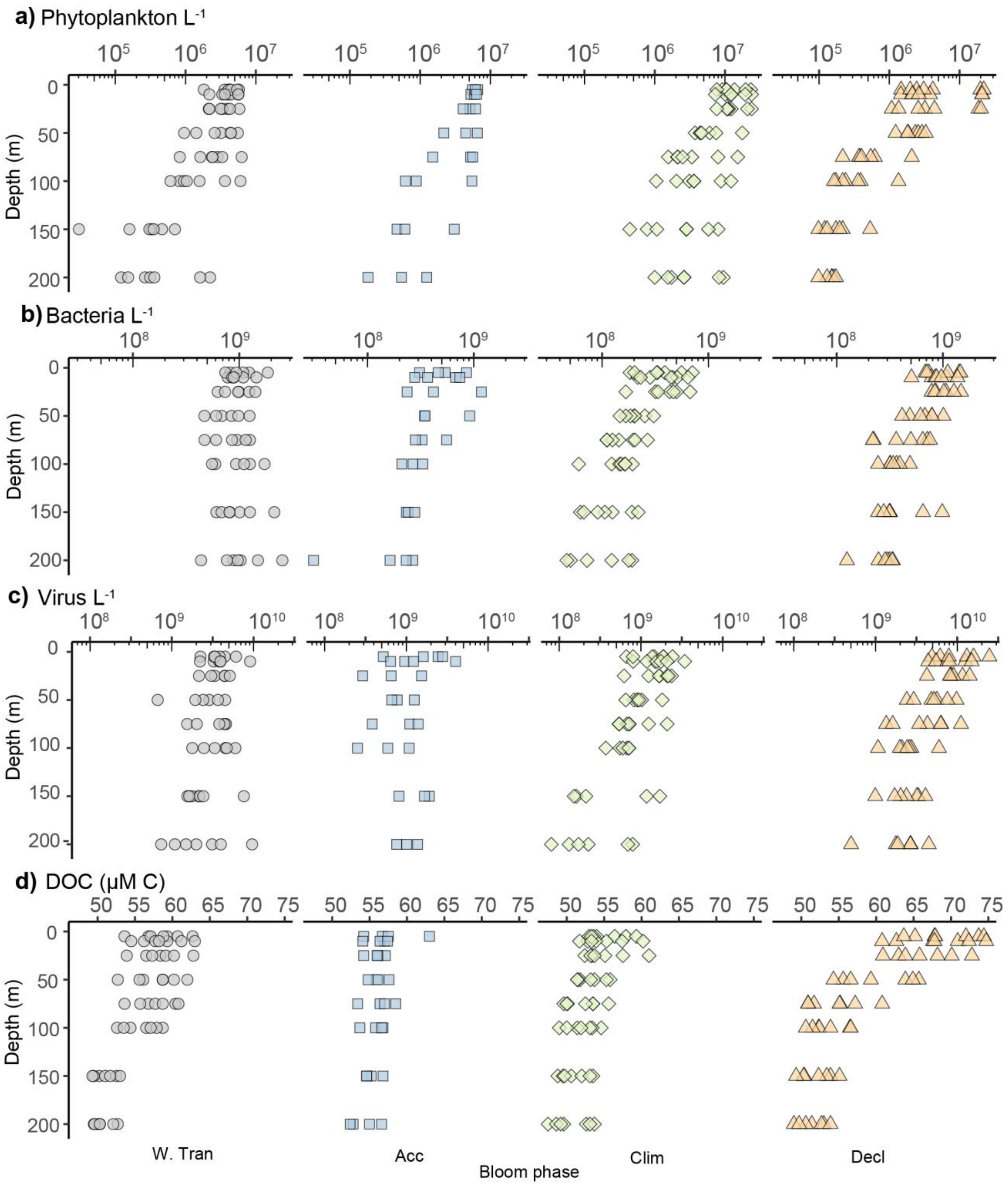

Supplementary Figure 8: Seasonal bloom progression results in vertical stratification of phytoplankton, bacteria, viruses, and DOC concentrations. Depth profiles of a) phytoplankton L^{-1}, b) bacteria L^{-1}, c) virus L^{-1} and d) dissolved organic carbon (μM carbon) concentrations across the seasonal bloom phases of the Western North Atlantic. Symbols are colored and shaped by bloom phase and represent individual water samples taken at the same time of day in unique stations throughout each phase. Phase abbreviations (bottom row): W. Tran. = Winter transition, Acc = Accumulation, Clim = Climax, Decl = Decline.

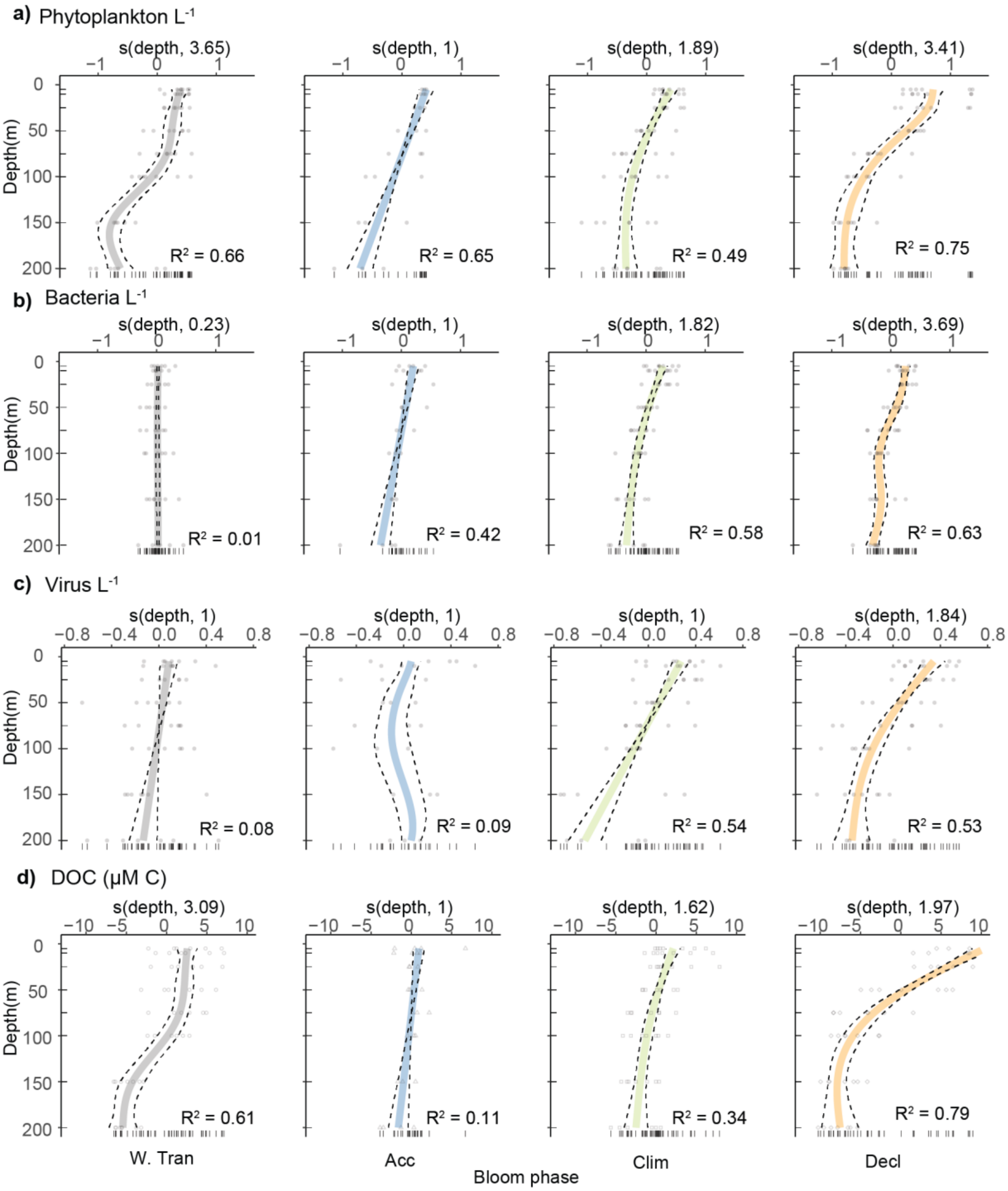

Supplementary Figure 9. Seasonal bloom progression results in vertical stratification of phytoplankton, bacteria, viruses, and DOC concentration. General additive modeling analysis for vertical profiles of a) phytoplankton cells L^{-1}, b) bacteria L^{-1}, c) viruses L^{-1}, and d) dissolved organic carbon (μM carbon). General additive modeling analysis fits a smoothing line on the data by optimizing the number of knots (i.e., connection) and setting the population mean to zero (x axis). Differences in each direction are the differences from the mean. When the fitted line is above/below the "0" value, the population mean at that depth can be considered enriched/depleted, respectively, compared to the rest of the depth profile. The number after depth in each panel represents the number of knots used for analysis. Colored and dotted lines

represent the fitted model and two standard errors. Spline types are defined in Supp. Table 1. Lines are colored by bloom phase. Small grey dots represent individual water samples taken at the same time of day in stations throughout each phase. Bloom phase abbreviations (bottom row): W. Tran = Winter transition, Acc = Accumulation, Clim = Climax, Decl = Decline. Number of independent samples, significance of smooth terms, and spline number input can be found in Source Data file.

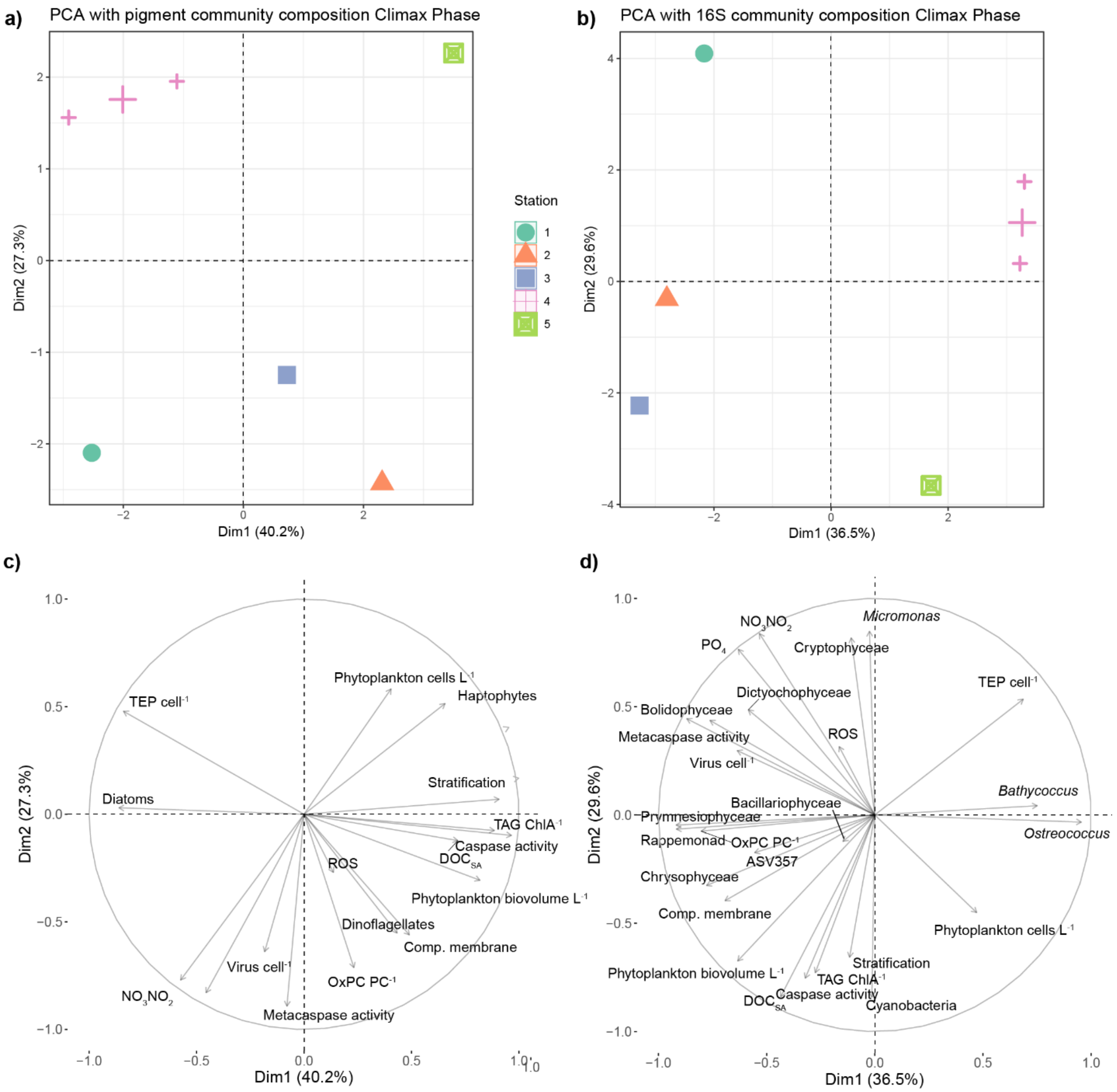

Supplementary Figure 10. Principle component analysis associates distinct physiological states, extracellular signatures, and phytoplankton community compositions within the Climax phase. (Top row) PCA showing interrelationships among measured intra- and extra-cellular biomarkers with phytoplankton populations using either a) pigment-based community composition[33] or b) 16S rRNA-based community composition[34]. Symbols are colored and shaped by station. Small symbols represent the median value for daily sampling events between 0500h and 0900h. Percent variability in each dimension is the amount of variance explained within the dataset. (Bottom row) PCA scores and vectors for measured intra- and extra-cellular properties with respect to (c) Pigment-based community composition and (d) 16S rRNA-based community composition. Phytoplankton cells L^{-1} = phytoplankton cell concentration; ROS = reactive oxygen species (fold change fluorescence from unstained); Comp. membrane = % of SYTOX positive cells; TEP $cell^{-1}$ = transparent exopolymer particle concentration (µg XG

eq. L^{-1}) normalized to bacteria and phytoplankton concentration; Virus $cell^{-1}$ = virus concentration normalized to bacteria and phytoplankton concentration; OxPC PC^{-1} = oxidized phosphatidylcholine (pm) normalized to total phosphatidylcholine; TAG = triacylglycerol (pM) normalized to ChlA; Caspase activity = caspase activity (µmol caspase substrate cleaved µg $protein^{-1}$ h^{-1}); Metacaspase activity = metacaspase activity (µmol metacaspase substrate cleaved µg $protein^{-1}$ h^{1}); DOC_{SA} = seasonally accumulated dissolved organic carbon (see Methods); Phytoplankton volume L^{-1} = phytoplankton µl L^{-1}; Stratification = stratification of the upper 300 m, measured as buoyancy frequency (s^{-1}). Longer arrows represent a stronger correlation with each respective dimension. Phytoplankton taxonomic groups were excluded from PCA analysis if no community type was detected within this bloom phase on the days sampled, or if no representative clade was identified.

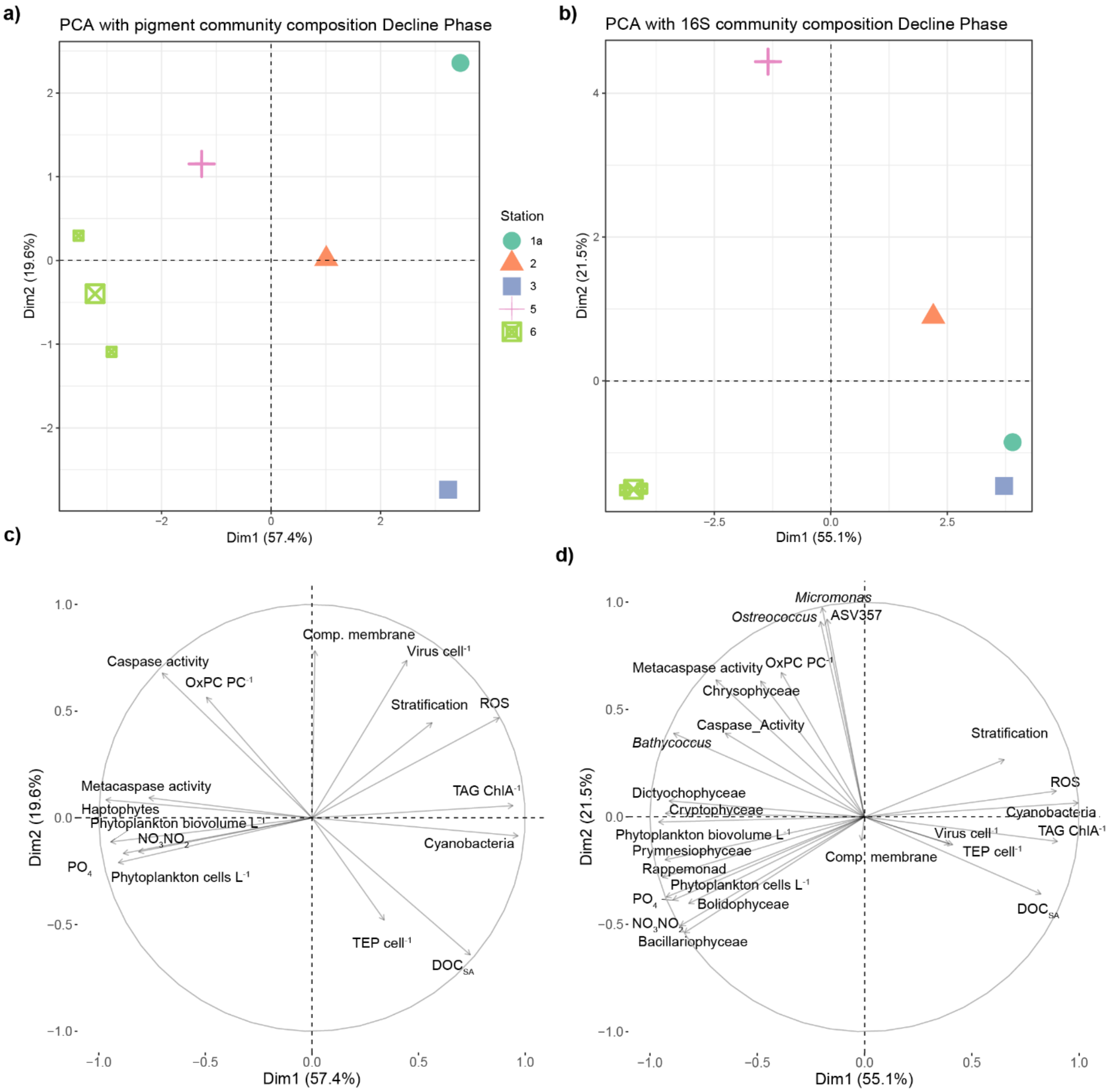

Supplementary Figure 11. Principle component analysis associates distinct physiological states, extracellular signatures, and phytoplankton community compositions within the Decline phase. (Top row) PCA showing interrelationships among measured intra- and extra-cellular biomarkers with phytoplankton populations using either a) pigment-based community composition[33] or b) 16S rRNA-based community composition[34]. Symbols are colored and shaped by station. Small symbols represent the median value for daily sampling events between 0500h and 0900h. Percent variability in each dimension is the amount of variance explained within the dataset. (Bottom row) PCA scores and vectors for measured intra- and extra-cellular properties with respect to (c) Pigment-based community composition and (d) 16S rRNA-based community composition. Phytoplankton cells L^{-1} = phytoplankton cell concentration; ROS = reactive oxygen species (fold change fluorescence from unstained); Comp. membrane = % of SYTOX positive cells; TEP $cell^{-1}$ = transparent exopolymer particle concentration (µg XG eq. L^{-1}) normalized to bacteria and phytoplankton concentration; Virus $cell^{-1}$ = virus

concentration normalized to bacteria and phytoplankton concentration; OxPC PC^{-1} = oxidized phosphatidylcholine (pm) normalized to total phosphatidylcholine; TAG = triacylglycerol (pM) normalized to ChlA; Caspase activity = caspase activity (µmol caspase substrate cleaved µg $protein^{-1}$ h^{-1}); Metacaspase activity = metacaspase activity (µmol metacaspase substrate cleaved µg $protein^{-1}$ h^{1}); DOC_{SA} = seasonally accumulated dissolved organic carbon (see Methods); Phytoplankton volume L^{-1} = phytoplankton µl L^{-1}; Stratification = stratification of the upper 300 m, measured as buoyancy frequency (s^{-1}). Longer arrows represent a stronger correlation with each respective dimension. Phytoplankton taxonomic groups were excluded from PCA analysis if no community type was detected within this bloom phase on the days sampled, or if no representative clade was identified.

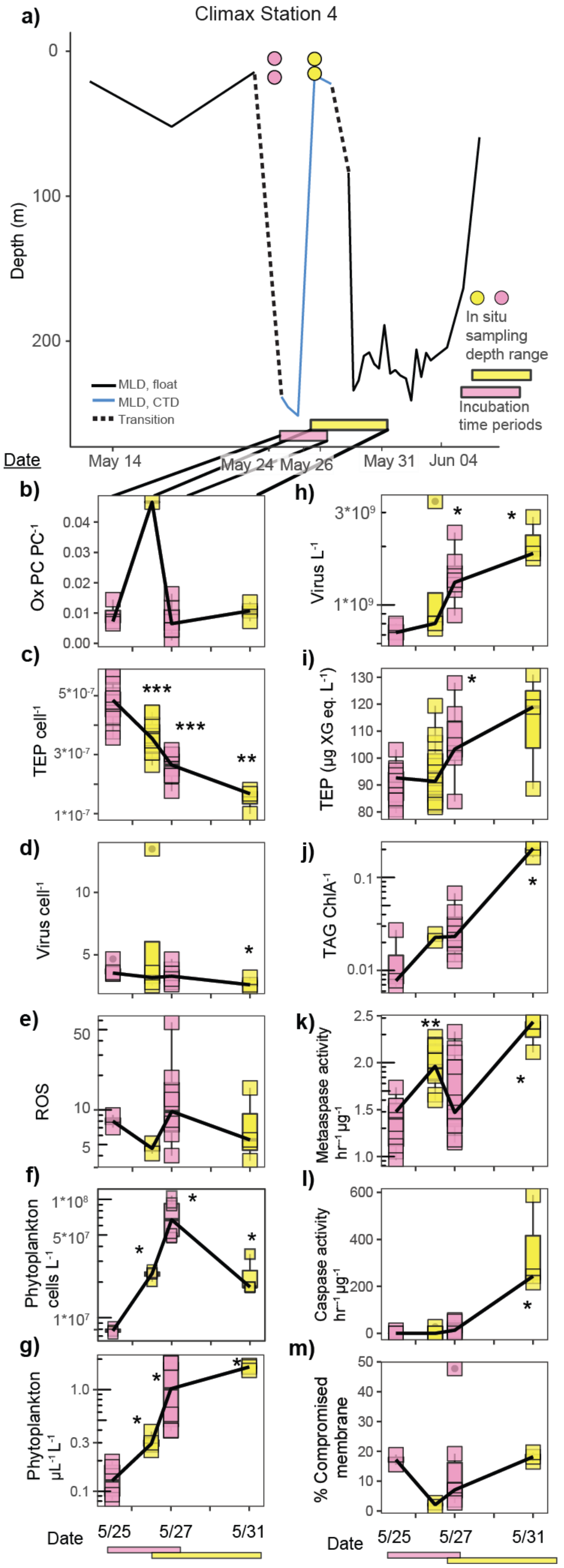

Supplementary Figure 12. Physiological state of phytoplankton transitioning from a deep to a

shallow mixed layer. a) Mixed layer depth (MLD) dynamics at Station 4 during the Climax phase in relation to sampled phytoplankton communities. Pre- and post-occupation MLDs are provided by drifting BioArgo floats (solid black line). A dashed line represents the transition from the float-measured MLD to ship-measured MLD (solid blue line), since sampling was done near and not directly at the float. Water sampled from the 1st and 3rd days of occupation is indicated by pink and yellow symbols, respectively. Colored circles represent sampling depths of *in situ* populations and correspond to between 5 and 25 m depth with time on x-axis corresponding to incubation times of *in situ* sampled communities (colored circles were the source water). Data for Day 4 and Day 8 incubations derive from the same *in situ* water collected on Day 1 and Day 3, respectively, and incubated on deck at *in situ* light and temperature (see Methods). Dates and data corresponding to these *in situ* sampling dates and final incubation times (b-l), and correspond to Figure 6. (b-m) Time course of measured biomarkers for *in situ* and incubated populations: b) Ox PC PC^{-1}= oxidized phosphatidylcholine (pm) normalized to total PC (pm); c) TEP cell^{-1}, transparent exopolymer particles normalized to phytoplankton and bacterial cells; d) Virus Cell^{-1} = virus concentration normalized to sum of phytoplankton and bacteria concentrations; e) ROS = reactive oxygen species, fold change from unstained; f) Phytoplankton cells L^{-1} = phytoplankton cell concentration; g) Phytoplankton µl L^{-1}= phytoplankton biovolume; h) Virus L^{-1} = virus concentration; i) TEP L^{-1}(µg XG eq. L^{-1}) = transparent exopolymer particles, xanthan gum equivalent; j) TAG ChlA^{-1} = triacylglycerol (pM) normalized to chlorophyll A (peak area); k) Metacaspase activity = µmol metacaspase substrate cleaved µg protein^{-1} hr^{1}; l) Caspase activity = µmol caspase substrate cleaved µg protein^{-1} hr^{-1}; m) % Compromised membrane = % of population with compromised membranes. Incubation samples from depths greater than 5 meters on 5/31 were lost due to a storm knocking the incubation tanks off the deck. Statistical analysis is not robust (n<3 for at least one day) for ROS, compromised membranes, and OxPC, due to loss of samples in transit on Day 1. Asterisks indicate significant differences from Day 1 via Kruskal Wallis test (* = p<0.05, ** = p<0.01, *** = p<0.001). Box plots represent the median value bounded by the upper and lower quartiles with whiskers representing the median + quartile*1.5. Number of biologically independent samples per sampling date (from left to right) = 3, 1, 6, 3 (b), 12, 12, 6, 3 (c), 4, 4, 6, 3 (d), 2, 2, 6, 3 (e), 4, 4, 6, 3 (f) 4, 4, 6, 3 (g), 4, 4, 6, 3 (h), 12, 12, 6, 3 (i), 4, 3, 8, 3 (j), 9, 8, 11, 3 (k), 9, 8, 11, 3 (l) 9, 8, 11, 3 (m). Exact p values found in Source Data file.

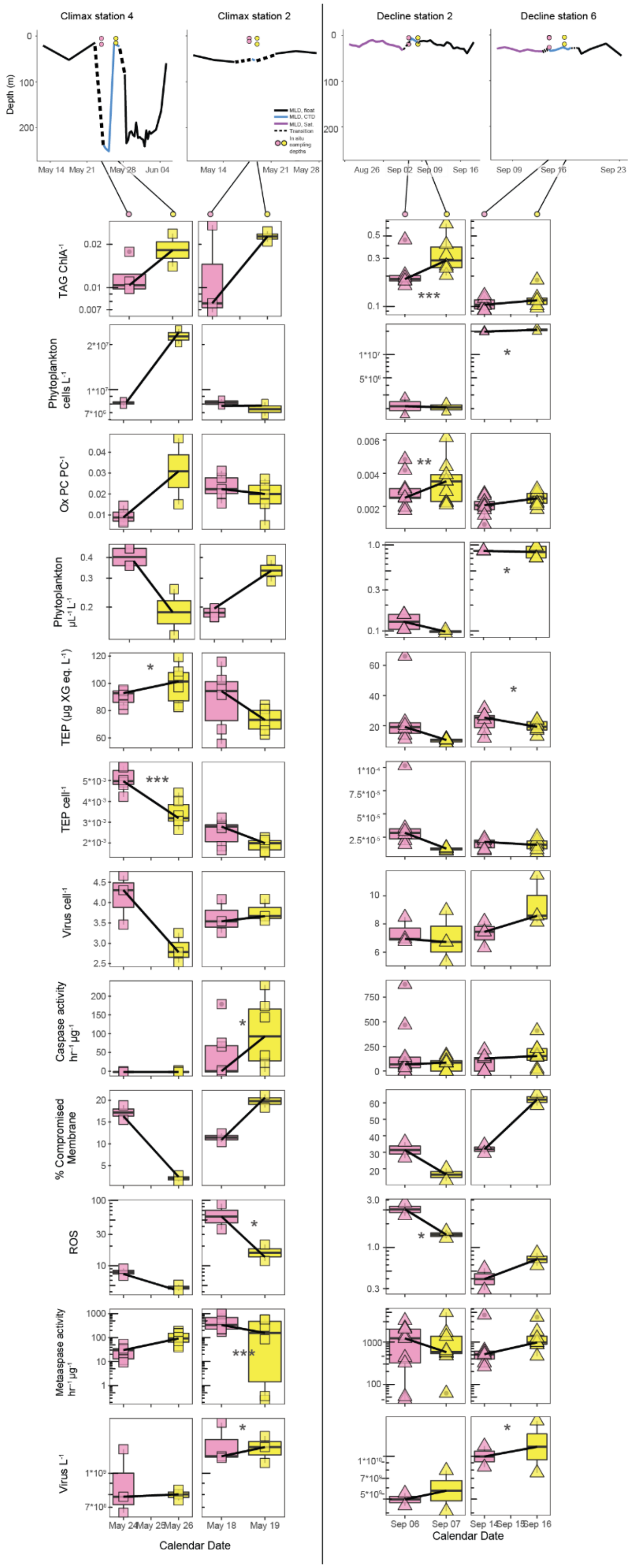

Supplementary Figure 13. Inter-station comparisons of phytoplankton physiological state in

water columns with different mixing dynamics and biomass loads. (Top panels) Mixed layer depth (MLD) dynamics at a subset of Climax and Decline phase stations in relation to sampled phytoplankton communities. Climax Station 4 transitioned from a deeply mixed to a shallow mixing water column, while Climax station 1 had more consistent MLDs during sample occupation. More stable and shallow MLDs were observed during the Decline phase, but phytoplankton communities had different relative biomass levels. Pre- and post-occupation MLDs are provided by drifting BioArgo floats (solid black line) and derived from satellite measurements (purple line), since BioArgo floats were not available for the Decline phase cruise (see Methods). A dashed line represents the transition from the float-measured MLD to ship-measured MLD (solid blue line), since sampling was done near and not directly at the float. Y-axis positions of colored circles represent sampling depths of *in situ* populations and correspond to 40% and 20% surface irradiance. X-axis position of colored circles represents the date of sampling. (Bottom panels) Comparison of intra- and extra-cellular biomarkers for phytoplankton associated with *in situ* samples from above. Pink symbols= 1st day of occupation. Yellow symbols = subsequent day of occupation. Asterisks indicate significant differences between stations, via Kruskal Wallis test (* = $p<0.05$, ** = $p<0.01$, *** = $p<0.001$). TAG ChlA^{-1} = triacylglycerol (pM) normalized to chlorophyll A (peak area); Phytoplankton cells L^{-1}= phytoplankton cell concentration; OxPC PC^{-1} = oxidized phosphatidylcholine (pm) normalized to total OxPC (pm); phytoplankton µl L^{-1}= phytoplankton biovolume; TEP L^{-1} (µg XG eq. L^{-1}); transparent exopolymer particles, xanthan gum equivalent; TEP cell^{-1} = transparent exopolymer particle concentration (µg XG eq. L^{-}1) normalized to phytoplankton and bacteria concentration; Virus cell^{-1} = virus concentrations normalized to phytoplankton and bacteria concentration; Caspase activity (hr^{-1} µg^{-1}) = µmol caspase substrate cleaved hour^{-1} µg protein^{-1}; % Compromised membrane = % of population with compromised membranes; ROS = reactive oxygen species, fold change from unstained; Metacaspase activity (hr^{-1} µg^{-1}) = µmol metacaspase substrate cleaved hour^{-1} µg protein^{-1}; Virus L^{-1} = virus concentration. Data from the climax phase stations were pooled from additional 1% irradiance depth samples due to some samples being lost in transit. Box plots represent the median value bounded by the upper and lower quartiles, with whiskers representing the median + quartile*1.5. Number of biologically independent samples per station/sampling date (from left to right) = 4, 2, 3, 2, 6, 6, 6, 6 (TAG), 2, 2, 2, 2, 2, 2, 2, 2 (Phytoplankton cells L^{-1}), 6, 6, 6, 3, 6, 6, 6, 6 (OxPC), 2, 2, 2, 2, 2, 2, 2, 2 (Phytoplankton µL^{-1}), 6, 6, 6, 6, 6, 6, 6, 6 (TEP L^{1}), 6, 6, 6, 6, 6, 6, 6, 6 (TEP cell^{1}), 2, 2, 2, 2, 2, 2, 2, 2 (Virus cell^{-1}), 6, 3, 6, 6, 6, 6, 5, 5 (Caspase activity), 2, 2, 2, 2, 2, 2, 2, 2 (% Comp. membrane), 2, 2, 2, 2, 2, 2, 2, 2 (% Comp. membrane), 6, 3, 6, 6, 6, 6, 5, 5 (Metacaspase activity), 2, 2, 2, 2, 2, 2, 2, 2 (Virus L^{-1}). Exact p value's can be found in the Source Data file.

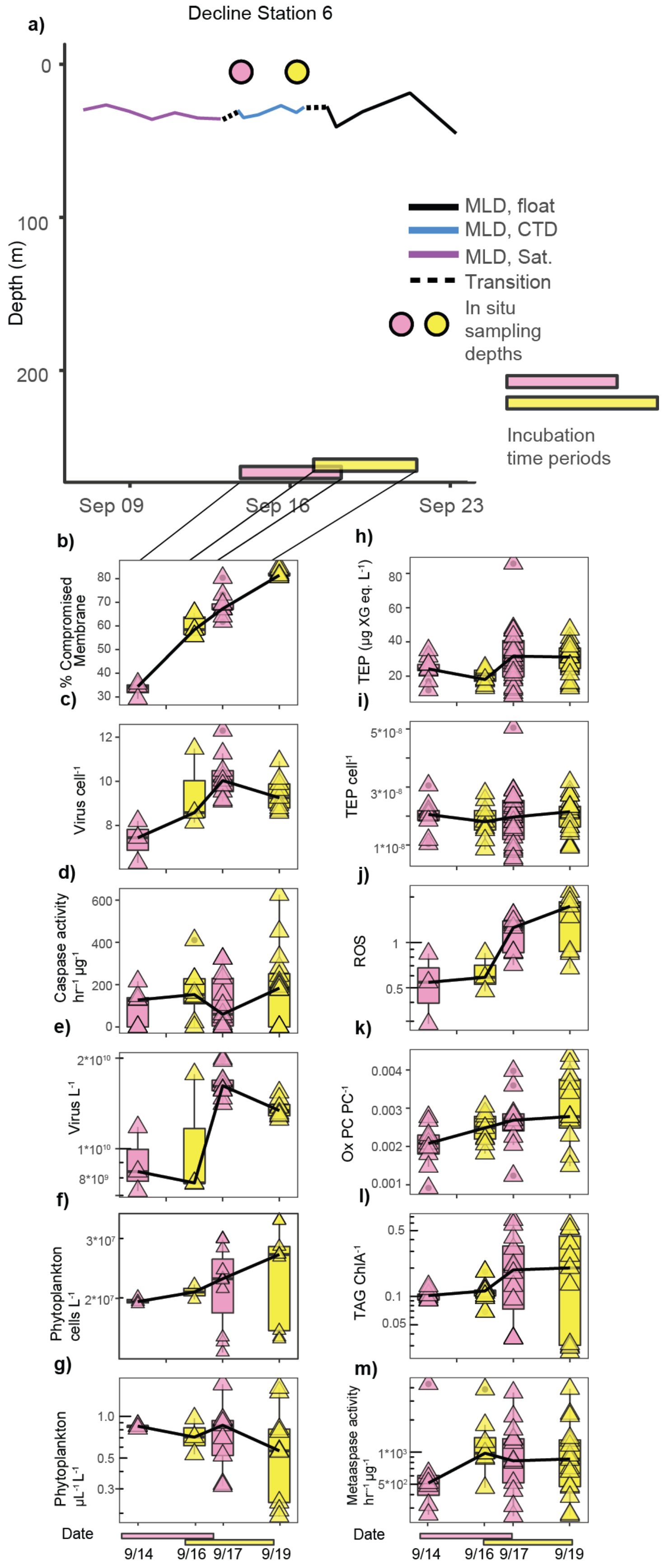

Supplementary Figure 14. Physiological state of a high biomass station observed during the

Decline phase. a) Mixed layer dynamics at Station 6 during the Decline phase expedition in relation to sampled phytoplankton communities. Pre- and post-occupation mixed layer depths (MLD) are provided by drifting BioArgo floats (solid black line) and derived from satellite measurements (purple line), since BioArgo floats were not available for the decline phase cruise (see Methods). A dashed line represents the transition from the float-measured MLD to ship-measured MLD (solid blue line), since sampling was done near and not directly at the float. Water sampled from the 1st and 3rd days of occupation is indicated by pink and yellow symbols, respectively. Colored circles represent sampling depths of *in situ* populations and correspond to 40% and 20% surface irradiance, with colored bars on time x-axis corresponding to incubation times of sampled communities (colored circles were the source water). Data for the second two time points were taken from the same water as 9/14 and 9/16, respectively, and incubated on deck at *in situ* light and temperature (see Methods). Dates and data corresponding to these *in situ* sampling dates and final incubation times are used. (b-m) Time course of measured biomarkers for *in situ* and incubated populations (indicated on x-axis): b) % compromised membrane = % of population with compromised membranes; c) virus cell^{-1} = virus concentration normalized to phytoplankton and bacteria concentration; d) caspase activity = µmol metacaspase substrate cleaved µg protein^{-1} h^{1} ; e) virus L^{-1} = virus concentration; f) phytoplankton cells L^{-1}= phytoplankton cell concentration; g) phytoplankton µl L^{-1}= phytoplankton biovolume; h) TEP L-1 (ng XG eq. L^{-1}) = transparent exopolymer particles, xanthan gum equivalent; i) TEP cell^{-1} = transparent exopolymer particle concentration (ng XG eq. L^{-1}) normalized to phytoplankton and bacteria concentration; j) ROS = reactive oxygen species, fold change from unstained; k) Ox PC PC^{-1} = oxidized phosphatidylcholine (pm) normalized to total PC (pm); l) TAG ChlA^{-1} = triacylglycerol (pM) normalized to chlorophyll A (peak area); m) metacaspase activity = µmol caspase substrate cleaved µg protein^{-1} hour^{-1}. Box plots represent the median value bounded by the upper and lower quartiles, with whiskers representing the median + quartile*1.5. Number of biologically independent samples per sampling date (from left to right) = 3, 3, 9, 9 (b), 3, 3, 9, 9 (c), 7, 8, 13, 16 (d), 3, 3, 9, 8 (e), 3, 2, 11, 9 (f), 3, 3, 9, 9 (g), 9, 9, 23, 22 (h), 9, 9, 24, 24 (i), 3, 3, 9, 9 (j), 9, 9, 11, 11 (k), 9, 9, 12, 11 (l), 7, 8, 13, 16 (m).

Supplementary References

1. Steele, D. J., Kimmance, S. A., Franklin, D. J. & Airs, R. L. Occurrence of chlorophyll allomers during virus-induced mortality and population decline in the ubiquitous picoeukaryote *Ostreococcus tauri*. *Environ. Microbiol.* 20, 588–601 (2018).
2. Schieler, B. M. *et al.* Nitric oxide production and antioxidant function during viral infection of the coccolithophore Emiliania huxleyi. *ISME J.* 13, 1019–1031 (2019).
3. Carrara, F., Sengupta, A., Behrendt, L., Vardi, A. & Stocker, R. Bistability in oxidative stress response determines the migration behavior of phytoplankton in turbulence. *Proc. Natl. Acad. Sci. U. S. A.* 118, (2021).
4. Hakkila, K. *et al.* Oxidative stress and photoinhibition can be separated in the cyanobacterium Synechocystis sp. PCC 6803. *Biochim. Biophys. Acta - Bioenerg.* 1837, 217–225 (2014).
5. Oparka, M. *et al.* Quantifying ROS levels using CM-H2DCFDA and HyPer. *Methods* vol. 109 3–11 (2016).
6. Dananjaya, S. H. S., Godahewa, G. I., Jayasooriya, R. G. P. T., Lee, J. & De Zoysa, M. Antimicrobial effects of chitosan silver nano composites (CAgNCs) on fish pathogenic Aliivibrio (Vibrio) salmonicida. *Aquaculture* 450, 422–430 (2016).
7. Rychtecký, P., Znachor, P. & Nedoma, J. Spatio-temporal study of phytoplankton cell viability in a eutrophic reservoir using SYTOX Green nucleic acid stain. *Hydrobiologia* 740, 177–189 (2014).
8. Brussaard, C., Marie, D., Thyrhaug, R. & Bratbak, G. Flow cytometric analysis of phytoplankton viability following viral infection. *Aquat. Microb. Ecol.* 26, 157–166 (2001).
9. Chapman, I. J., Esteban, G. F. & Franklin, D. J. Molecular probe optimization to determine cell mortality in a photosynthetic organism (Microcystis aeruginosa) using flow cytometry. *J. Vis. Exp.* 2016, 53036 (2016).
10. Gerphagnon, M., Latour, D., Colombet, J. & Sime-Ngando, T. A double staining method using SYTOX green and calcofluor white for studying fungal parasites of phytoplankton. *Appl. Environ. Microbiol.* 79, 3943–3951 (2013).
11. Lebaron, P., Catala, P. & Parthuisot, N. Effectiveness of SYTOX green stain for bacterial viability assessment. *Appl. Environ. Microbiol.* 64, 2697–2700 (1998).
12. Becker, K. W. *et al.* Daily changes in phytoplankton lipidomes reveal mechanisms of energy storage in the open ocean. *Nat. Commun.* 9, 1–9 (2018).
13. Lee, R., Hagen, W. & Kattner, G. Lipid storage in marine zooplankton. *Mar. Ecol. Prog. Ser.* 307, 273–306 (2006).
14. Collins, J. R. *et al. The molecular products and biogeochemical significance of lipid photooxidation in West Antarctic surface waters*.
15. Bochkov, V. N. *et al.* Generation and biological activities of oxidized phospholipids. *Antioxidants and Redox Signaling* vol. 12 1009–1059 (2010).
16. Bidle, K. D. The Molecular Ecophysiology of Programmed Cell Death in Marine Phytoplankton. *Ann. Rev. Mar. Sci.* 7, 341–375 (2015).
17. Seth-Pasricha, M., Bidle, K. A. & Bidle, K. D. Specificity of archaeal caspase activity in the extreme halophile Haloferax volcanii. *Environ. Microbiol. Rep.* 5, 263–271 (2013).
18. Asplund-Samuelsson, J. *et al.* Diversity and expression of bacterial metacaspases in an aquatic ecosystem. *Front. Microbiol.* 7, 1043 (2016).
19. Brussaard, C. P. D. Optimization of Procedures for Counting Viruses by Flow Cytometry. *Appl. Environ. Microbiol.* 70, 1506–1513 (2004).
20. Cooper, W., Lirman, D., Schmale, M. & Lipscomb, D. Consumption of coral spat by histophagic ciliates. *Coral Reefs* 26, 249–250 (2007).
21. Duhamel, S. & Jacquet, S. Flow cytometric analysis of bacteria- and virus-like particles in

lake sediments. *J. Microbiol. Methods* 64, 316–332 (2006).
22. Grossart, H. P., Czub, G. & Simon, M. Algae-bacteria interactions and their effects on aggregation and organic matter flux in the sea. *Environ. Microbiol.* 8, 1074–1084 (2006).
23. Nissimov, J. I. *et al.* Dynamics of transparent exopolymer particle production and aggregation during viral infection of the coccolithophore, Emiliania huxleyi. *Environ. Microbiol.* 20, 2880–2897 (2018).
24. Lønborg, C., Middelboe, M. & Brussaard, C. P. D. Viral lysis of Micromonas pusilla: Impacts on dissolved organic matter production and composition. *Biogeochemistry* 116, 231–240 (2013).
25. Toullec, J. & Moriceau, B. Transparent Exopolymeric Particles (TEP) Selectively Increase Biogenic Silica Dissolution From Fossil Diatoms as Compared to Fresh Diatoms. *Front. Mar. Sci.* 5, 102 (2018).
26. Thornton, D. C. O. & Chen, J. Exopolymer production as a function of cell permeability and death in a diatom (Thalassiosira weissflogii) and a cyanobacterium (Synechococcus elongatus). *J. Phycol.* (2017) doi:10.1111/jpy.12470.
27. Cruz, B. N. & Neuer, S. Heterotrophic bacteria enhance the aggregation of the marine picocyanobacteria prochlorococcus and synechococcus. *Front. Microbiol.* 10, (2019).
28. Della Penna, A. & Gaube, P. Overview of (sub)mesoscale ocean dynamics for the NAAMES field program. *Front. Mar. Sci.* 6, 384 (2019).
29. Behrenfeld, M. J. *et al.* The North Atlantic Aerosol and Marine Ecosystem Study (NAAMES): Science Motive and Mission Overview. *Front. Mar. Sci.* 6, (2019).
30. Baetge, N. *et al.* The Seasonal Flux and Fate of Dissolved Organic Carbon Through Bacterioplankton in the Western North Atlantic. *Front. Microbiol.* 12, (2021).
31. Fukuda, R., Ogawa, H., Nagata, T. & Koike, I. Direct determination of carbon and nitrogen contents of natural bacterial assemblages in marine environments. *Appl. Environ. Microbiol.* 64, 3352–3358 (1998).
32. Jover, L. F., Effler, T. C., Buchan, A., Wilhelm, S. W. & Weitz, J. S. The elemental composition of virus particles: Implications for marine biogeochemical cycles. *Nat. Rev. Microbiol.* 12, 519–528 (2014).
33. Kramer, S. J., Siegel, D. A. & Graff, J. R. Phytoplankton Community Composition Determined From Co-variability Among Phytoplankton Pigments From the NAAMES Field Campaign. *Front. Mar. Sci.* 7, (2020).
34. Bolaños, L. M. *et al.* Seasonality of the Microbial Community Composition in the North Atlantic. *Front. Mar. Sci.* 8, 23 (2021).
