# Supplementary material for: Seasonal mixed layer depth shapes phytoplankton physiology, viral production, and accumulation in the North Atlantic
Source: Nat Commun. 2021 Nov 17;12:6634. doi: 10.1038/s41467-021-26836-1 (PMC8599477; doi:10.1038/s41467-021-26836-1)
Supplement: Supplementary file 3 — Reporting Summary [file 41467_2021_26836_MOESM3_ESM.pdf]

nature research

Corresponding author(s): Kay Bidle

Last updated by author(s): 9/20/2021

Reporting Summary

Nature Research wishes to improve the reproducibility of the work that we publish. This form provides structure for consistency and transparency in reporting. For further information on Nature Research policies, see our Editorial Policies and the Editorial Policy Checklist.

Statistics

For all statistical analyses, confirm that the following items are present in the figure legend, table legend, main text, or Methods section.

n/a	Confirmed	
☐	☒	The exact sample size (*n*) for each experimental group/condition, given as a discrete number and unit of measurement
☐	☒	A statement on whether measurements were taken from distinct samples or whether the same sample was measured repeatedly
☐	☒	The statistical test(s) used AND whether they are one- or two-sided<br>*Only common tests should be described solely by name; describe more complex techniques in the Methods section.*
☒	☐	A description of all covariates tested
☐	☒	A description of any assumptions or corrections, such as tests of normality and adjustment for multiple comparisons
☐	☒	A full description of the statistical parameters including central tendency (e.g. means) or other basic estimates (e.g. regression coefficient) AND variation (e.g. standard deviation) or associated estimates of uncertainty (e.g. confidence intervals)
☐	☒	For null hypothesis testing, the test statistic (e.g. *F*, *t*, *r*) with confidence intervals, effect sizes, degrees of freedom and *P* value noted<br>*Give P values as exact values whenever suitable.*
☒	☐	For Bayesian analysis, information on the choice of priors and Markov chain Monte Carlo settings
☒	☐	For hierarchical and complex designs, identification of the appropriate level for tests and full reporting of outcomes
☒	☐	Estimates of effect sizes (e.g. Cohen's *d*, Pearson's *r*), indicating how they were calculated

Our web collection on statistics for biologists contains articles on many of the points above.

Software and code

Policy information about availability of computer code

Data collection	Enumeration via flow cytometry (was gated in FlowJo v7 from cytograms generated via flow cytometry using BD flow cytometry software (Accuri C6, Influx). Lipid data data was processed with the Thermo Scientific Sieve software. Caspase activity rates (including protein concentration) were collected with Spectramax M3 spectrophotometer and exported to Excel from the Softmax Pro 6.3 software.
Data analysis	All statistics and plotting were done in R - general visualization done with the ggplot; General Additive Modeling of biomarkers within depth profiles was done using the mgcv package in R, while smoothing to represent distributions between bloom phases was done with the ggridges package.

For manuscripts utilizing custom algorithms or software that are central to the research but not yet described in published literature, software must be made available to editors and reviewers. We strongly encourage code deposition in a community repository (e.g. GitHub). See the Nature Research guidelines for submitting code & software for further information.

Data

Policy information about availability of data

All manuscripts must include a data availability statement. This statement should provide the following information, where applicable:

- Accession codes, unique identifiers, or web links for publicly available datasets
- A list of figures that have associated raw data
- A description of any restrictions on data availability

Raw datasets of measured environmental parameters during the NAAMES cruises are accessible in the NASA SeaBass archive (https://seabass.gsfc.nasa.gov). Analyzed and quality-controlled datasets of environmental and physiological measurements will be made available on GitHub upon publication.

Field-specific reporting

Please select the one below that is the best fit for your research. If you are not sure, read the appropriate sections before making your selection.

☐ Life sciences ☐ Behavioural & social sciences ☒ Ecological, evolutionary & environmental sciences

For a reference copy of the document with all sections, see nature.com/documents/nr-reporting-summary-flat.pdf

Ecological, evolutionary & environmental sciences study design

All studies must disclose on these points even when the disclosure is negative.

Study description	We analyzed the physiology of phytoplankton in the North Atlantic with a suite of intra -and extra-cellular biomarkers. We assessed the differences in physiology between four bloom phases. We were interested in what shaped variation in these biomarkers within a specific bloom phase and specifically in relation to changes in mixed layer depths across seasons and even within a population/ location (e.g., Climax Station 4).
Research sample	We targeted a range of eukaryotic phytoplankton cell sizes (1-20 microns), given our flow cytometer is able to accurately quantify this size range and it is representative of the range of eukaryotic phytoplankton cells typically found in natural populations in the North Atlantic. Similarly, we targeted bacteria and viruses in their typical respective size ranges (0.2 micron and less than 0.5 microns), which were also within our detection limits.
Sampling strategy	Intracellular and extracellular measurements had the following sample strategy: The suite of diagnostic staining and flow cytometry measurements were performed on 1 replicate per depth per station for in situ CTD casts and 1 replicate from each triplicate incubation bottle per depth per station; each incubation employed 3 replicates per sampling depth. Samples were processed and stained within 1 h of collection and subseuqently analyzed on BD Accuri instrument. For transparent exopolymer particles (TEP), caspase/metacaspase activity, and lipids, biomass was collected in triplicate onto membrane filters for each depth for in situ CTD casts and processed according to stated Methods; triplicate filters were also taken from each triplicate incubation bottle at each depth. Sample sizes (replicates; volumes filtered) were determined based on measurements (including degree of variability) from previous fieldwork in the North Atlantic.
Data collection	Live phytoplankton were counted by Jason Graff on the BD Influx Mariner (in situ accumulation rates), and counted and stained (incubation accumulation rates, ROS, SYTOX) by the Bidle lab members (Ben Diaz, Christien Laber, Ben Knowles, Chris Johns) on the BD Accuri C6 (Software version 1.0.264.21). Biomass was filtered and frozen (caspase, metacaspase, and lipids), seawater glutaraldehyde-fixed and frozen (virus and bacteria counts), and TEP filtered, stained and frozen by Ben Diaz, Christien Laber, Ben Knowles, Chris Johns, Kay Bidle, and Elizabeth Harvey. TEP, metacaspase and caspase activity were obtained on a Molecular Devices SpectraMax M3 spectrophotometer (SoftMax Pro version 6.3 software) by Liti Haramatay and Ben Diaz. Virus and bacteria counts were collected from fixed seawater by Chris Johns, Frank Natale, and Ben Diaz on the BD Influx Mariner. Seawater was filtered for DOC analysis by Nick Baetge, and DOC was recorded with a Shimadzu TOC-V or TOC-L. Seawater was filtered for 16S community DNA analysis by Luis Bolanos and analyzed using amplicons and Illumina MiSeq. Seawater was filtered for HPLC pigment community analysis by Sasha Kramer and members of the NAAMES science team and processed via HPLC by Crystal Thomas at NASA GSFC. Lipid processing/ MS (HPLC/ESI HRAM MS) using an Agilent 1200 HPLC coupled to an HRAM MS, using LOBSTAHS v 3.1, Thermo Scientific Xcalibur v. 3.1) was done by Jonathon Hunter and Daniel Lowenstein.
Timing and spatial scale	In situ sampling occurred in four phases/ NAAMES expeditions. NAAMES I (Winter Transition) started 11/12/2015, and ended 11/25/2015. NAAMES II (Climax) started 5/17/2016 and ended 5/31/2016. NAAMES III (Decline) started 9/4/2017 and ended 9/17/2017. NAAMES IV (Accumulation) began 3/26/2018 and ended on 4/5/2018. Between 4-7 stations were visited each expedition. The study region was bound between 40°N and 55°N, and was along 40°W longitude. The rationale for this temporal and geographic sampling strategy was to capture populations at distinct annual bloom phases and across different geographical locations (distinct populations; water masses). Sampling was performed once per day in the morning (two hours pre-dawn to avoid the influence of variable light on populations at the time of sampling), over the course of a two hours--the time it took to deploy and recover the CTD rosette instruments). Beginning from NAAMES I, optical float profilers were deployed and continuously monitored the mixed layer depth throughout the study region. This data provided information for an adaptive sampling strategy for targeted water masses for which mixed layer depths and dynamics had been characterized (a central focus of the study).
Data exclusions	For most of the article, samples that were below the mixed layer depth were excluded from analysis since we based our sampling depths on the euphotic zone depth, which lies completely within deep mixed layers, and outside of shallower mixed layers. In some bloom phases, this meant that we had very few samples from outside the mixed layer. Since other evidence suggests that phytoplankton outside of the mixed layer might undergo unique stresses from less light availability, we excluded these samples when where we had them. We also excluded a few samples from May 2016 when it appeared that the flow cytometer was jammed. This was evident due to non-standard flow rates and characteristic FSC-H "spikes". The clog went undetected, and apparently resolved itself a few days later.
Reproducibility	Our data derive from a suite of physiological measurements of natural phytoplankton populations (diagnostic staining, caspase/ metacaspase activity, lipids, etc) and associated extracellular signatures (TEP, DOC, viruses) along with environmental parameters (e.g., light, MLD, buoyancy frequency, etc) sampled across the North Atlantic during different seasons. To adequately assess the reproducibility of these parameters/measurements, they were sampled in triplicate at discrete depths (and light levels); furthermore, on-deck incubations used triplicate bottle incubations for each sampling condition (station/depth/light level) with triplicate samples also taken within each bottle. This adequately captured the reproducibility of processes measured across the different phytoplankton populations, water masses and seasons and revealed the degree of inherent variability.

Randomization	Given this was an observational study, whereby sampling was performed of specific phytoplankton populations at specific depths across different geographical locations over four seasonal bloom phases (as outlined in the NAAMES mission overview), randomization of samples across conditions/etc was not appropriate. No manipulative experimental treatments were performed. Rather, samples were maintained (for incubations) at the respective and appropriate light and temperature levels and measured accordingly.
Blinding	Given the location (and distinct depths) of samples collected in the water column were critical to sampling strategies, associated community physiology, and the implementation of on-deck incubations (including the use of relevant light levels for respective depths), sample blinding was not justified.

Did the study involve field work? ☒ Yes ☐ No

Field work, collection and transport

Field conditions	Field work was conducted in four bloom phases, which were defined on the basis of seasonal mixed layer dynamics and phytoplankton biomass via continuous remote observations and ship-based sampling. Relevant environmental parameters to the study question (and phytoplankton investigated) were: light (vertical distribution in water column); seawater temperature, salinity and density; mixed layer depth; stratification (buoyancy frequency) and inorganic macronutrient concentrations (N, P); dissolved organic carbon concentrations.
Location	The study region was in the Northwest Atlantic, bounded between 40°N and 55°N, and was along 40°W longitude.
Access & import/export	WHOI and the US Navy, who operate and own the R/V Atlantis, respectively, abide by applicable laws and regulations during the NAAMES expeditions. Scientists on-board including our lab group complied with the crew of the R/V Atlantis.
Disturbance	The only potential disturbance we are aware of is the pulse of phytoplankton and bacterial growth associated with off-boarding of the ship's lavatory waste. This was done in between sampling stations so our analysis does not reflect this potential disturbance.

Reporting for specific materials, systems and methods

We require information from authors about some types of materials, experimental systems and methods used in many studies. Here, indicate whether each material, system or method listed is relevant to your study. If you are not sure if a list item applies to your research, read the appropriate section before selecting a response.

Materials & experimental systems

n/a	Involved in the study
☒	☐ Antibodies
☒	☐ Eukaryotic cell lines
☒	☐ Palaeontology and archaeology
☒	☐ Animals and other organisms
☒	☐ Human research participants
☒	☐ Clinical data
☒	☐ Dual use research of concern

Methods

n/a	Involved in the study
☒	☐ ChIP-seq
☐	☒ Flow cytometry
☒	☐ MRI-based neuroimaging

Flow Cytometry

Plots

Confirm that:

☒ The axis labels state the marker and fluorochrome used (e.g. CD4-FITC).

☒ The axis scales are clearly visible. Include numbers along axes only for bottom left plot of group (a 'group' is an analysis of identical markers).

☒ All plots are contour plots with outliers or pseudocolor plots.

☒ A numerical value for number of cells or percentage (with statistics) is provided.

Methodology

Sample preparation	Water for sampling was collected from throughout the water column with a Niskin bottle on a CTD-rosette package, except for tw stations in NAAMES IV (Accumulation phase), where in-line Cells were subjected to varied infection treatments by the virus strain EhV207. Cell abundances and physiological state were assessed by flow cytomtery and viral abundances by qPCR. Viral and bacterial enumerations were made from glutaraldehyde-fixed (0.5% V/V) samples. Caspase activity was measured on extracts from frozen biomass filters via sonication in caspase reaction buffer (50 mM HEPES, 50 mM NaCl, 0.1% CHAPS, 10 mM EDTA, 5% glycerol, 10 mM dithiothreitol, pH 7.2).
Instrument	Data were collected on a custom BD Influx and Accuri C6 flow cytometers.

Software	Data were collected with BD Influx Mariner and Accuri software. Data were extracted from the resulting flow cytograms using FlowJo v7 and parsed into spreadsheets in Excel for plotting and analyses with R.
Cell population abundance	No sorts were done.
Gating strategy	Cells that were above 80,000 FSC-H and 1,000 FL3-H (670 LP emission from 488nm laser excitation) were counted as phytoplankton. Glutaldehyde-fixezd, SYBR-GOLD stained samples were gated based on a 520 nm emission threshold (via 488nm laser excitation) and classified as virus if they were below 0.2 microns, and as bacteria if they were below 0.5 micron and above 0.2 microns.

☒ Tick this box to confirm that a figure exemplifying the gating strategy is provided in the Supplementary Information.
